# Respiratory Distress: An Escape Room Simulation Case for Pediatric Care Providers

**DOI:** 10.7759/cureus.84851

**Published:** 2025-05-26

**Authors:** Karen Dermer, Sean O'Connor, Thomas Marchhart, Caitlin Keane-Bisconti, Rahul S Panesar

**Affiliations:** 1 Pediatrics, Stony Brook Children's Hospital, Stony Brook, USA; 2 Pediatric Critical Care, Stony Brook Children's Hospital, Stony Brook, USA; 3 Pediatric Emergency Medicine, Stony Brook Children's Hospital, Stony Brook, USA

**Keywords:** escape room simulation, pediatric cardiac arrest, pediatric residency education, pediatric respiratory distress, pediatric resuscitation, simulation in medical education

## Abstract

Escape room simulation has been proven to be an effective educational tool in adult medicine, but research on its use in pediatrics is lacking. Both escape room simulation and pediatric resuscitation require effective communication and critical thinking. Merging these two concepts can provide a novel way to teach trainees. We designed an escape room case to teach concepts of basic life support (BLS) and pediatric advanced life support (PALS) using a common pediatric presentation of respiratory distress leading to respiratory failure. Learners were required to use the skills and concepts from BLS and PALS as well as hospital resources to solve puzzles in the escape room. These puzzles were reflective of the team’s ability to recognize and treat the patient’s condition. In order to successfully escape the room, all puzzles needed to be solved in a timely manner. Learners were then surveyed on their experiences. Participants reported the simulation provided a valuable learning experience and supportive learning environment. They also endorsed an increase in medical knowledge and improvement of communication skills. Finally, escape room simulations were reported to be more engaging than typical mock codes.

## Introduction

The identification and initial stabilization of a critically ill child is a fundamental skill for any pediatrician as per the American Board of Pediatrics and the Accreditation Council of Graduate Medical Education [1-3]. However, in-house pediatric cardiopulmonary arrests are rare and opportunities to practice resuscitation are limited. As a result, pediatric trainees frequently endorse low confidence in their ability to perform in these scenarios [4,5]. Moreover, prior analyses of simulated pediatric emergencies have identified frequent communication errors, deviations from the American Heart Association's (AHA) Basic Life Support (BLS) protocols, and delays in care during the critical first five minutes of a cardiopulmonary arrest [6,7]. Similar studies have also reported an increase in trainee confidence and knowledge following participation in mock codes; however, these advances are not lasting and deteriorate quickly [8-10]. Escape rooms are a popular teaching modality for adult learners and have recently become of interest to healthcare educators given their emphasis on teamwork, communication, and critical thinking [11,12]. When used in medical education, most learners universally reported positive experiences with the escape room framework. Although this modality has been incorporated into adult medical education, its use for pediatric resident and nurse training is lacking. Additionally, pediatric trainees are usually the first responders to in-house codes, which warrants attention to the first five minutes of a cardiopulmonary arrest (e.g. ‘code blue’) [6]. We designed and trialed an escape room to emphasize the initial interventions needed in such pediatric emergencies. We then surveyed participants on their experiences. Our learning objectives were for learners to recognize a pediatric patient’s clinical deterioration and initiate a rapid response team, practice principles of crisis resource management (CRM), demonstrate BLS and pediatric advanced life support (PALS) skills, and endorse a positive learning experience with a new educational modality.

## Materials and methods

Development

We designed an escape room to be run in an actual patient room in our children’s hospital, an academic tertiary care center with 104 inpatient beds, pediatric emergency department (ED) volume of over 25,000 annually, 8,000 inpatient admissions annually, and the county’s only level 1 pediatric trauma center. This simulation was based on the children’s hospital acute care inpatient unit. We began running sessions in September 2023 through April 2024. The simulated nine-month-old patient presented with respiratory distress and progressed to respiratory failure resulting in cardiac arrest. Trainees were pre-briefed on escape room logistics and were required to have some knowledge of pediatric respiratory distress and its management. Learners needed to gather information about the patient and perform BLS skills including establishing an airway, providing bag mask ventilation, and compressions. Each concept was incorporated into a puzzle. Six puzzles were created in total, which needed to be solved to successfully ‘escape’ the room. The puzzles were designed to help learners identify respiratory distress in a baby, escalate care, and eventually begin cardiopulmonary resuscitation (CPR) and administer epinephrine. For example, in order to obtain a current set of vitals indicative of respiratory distress (hypoxia, tachypnea, and tachycardia), the participants needed to solve a jigsaw puzzle hanging from the cardiac monitor. Each time a puzzle was solved, a number was placed on a board located in the front of the room, with six slots corresponding to each puzzle. The placement and solutions of the puzzles also highlighted the principles of CRM. The order of the puzzles was designed to mimic the order of events that would ideally take place during a resuscitation. For example, epinephrine was not accessible to learners until all prior puzzles were solved, including placing defibrillator pads on the patient and a backboard beneath them. Access to the medication was blocked with a six-number combination lock that learners could only open after they solved all the puzzles and deduced that the numbers on the board were the code for the combination lock. Participants were given three ‘hint coupons’ to help advance them through the escape room if necessary. Facilitators were also provided scripts indicating actions to take if puzzles were not solved within a certain amount of time. Of note, the simulation always led to the patient decompensating into cardiac arrest. The purpose of this was to hone in on the learning objectives of recognition of a decompensating patient, initiation of a rapid response, proper use of CRM, and proper demonstration of BLS and PALS principles. This escape room simulation was conducted with a group of pediatric and medicine-pediatric residents from all levels of training and pediatric nurses. The team had 30 minutes to escape the room and were asked to complete a survey on their experience as part of the debrief.

Equipment/environment

**Figure 1:**
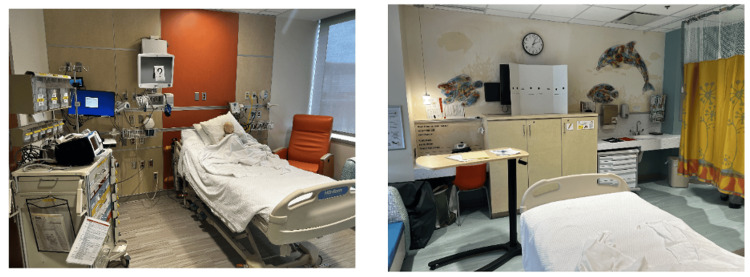
Room setup for escape room simulation in typical children's hospital patient room.

This case took place in one of the inpatient rooms in our children’s hospital. The simulation rotated in terms of location based on room availability. An infant mannequin was positioned in an upright hospital bed propped up on a pillow with a functioning peripheral IV. The simulated patient was alone in the room without a family member or bedside nurse to provide a background history. The room had a door note with the patient’s name and age, cardiac monitor, and fully stocked crash cart. Six escape room puzzles were scattered throughout the room (Figure 1).

Personnel

Chief pediatric residents, a pediatric critical care nurse, and/or pediatric subspecialty attendings (pediatric intensivists and/or pediatric emergency medicine) served as facilitators. Their responsibilities included orienting and debriefing learners and running the 30-minute escape room simulation in its entirety. Actors were not utilized for this case.

Implementation

**Figure 2:**
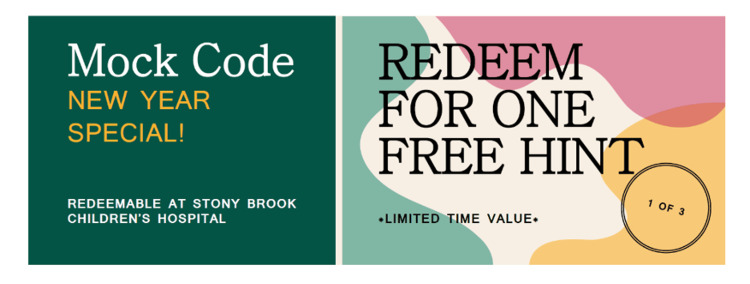
Hint Coupon

At least three pediatric and/or medicine-pediatric residents of all levels of training and one or two pediatric nurses were recruited for participation in the escape room simulation. Inclusion criteria included being a pediatric trainee (in either the pediatric or medicine-pediatric residency program), or a staff pediatric registered nurse. Exclusion criteria were limited to prior participation in the escape room simulation case. Participants were permitted to decline participation in the pre-brief if they chose. Sessions were conducted from September 2023 to April 2024. With this yearly variability and range of post-graduate training years, participant knowledge base ranged from that expected of a new intern to a senior resident. The facilitator(s) conducted a brief orientation on the escape room logistics (Appendix A). Learners were told they had 30 minutes to solve six puzzles to aid in the management and care of their simulated patient. Three hint coupons were provided if the learners found themselves stuck with solving a puzzle (Figure 2).

**Figure 3:**
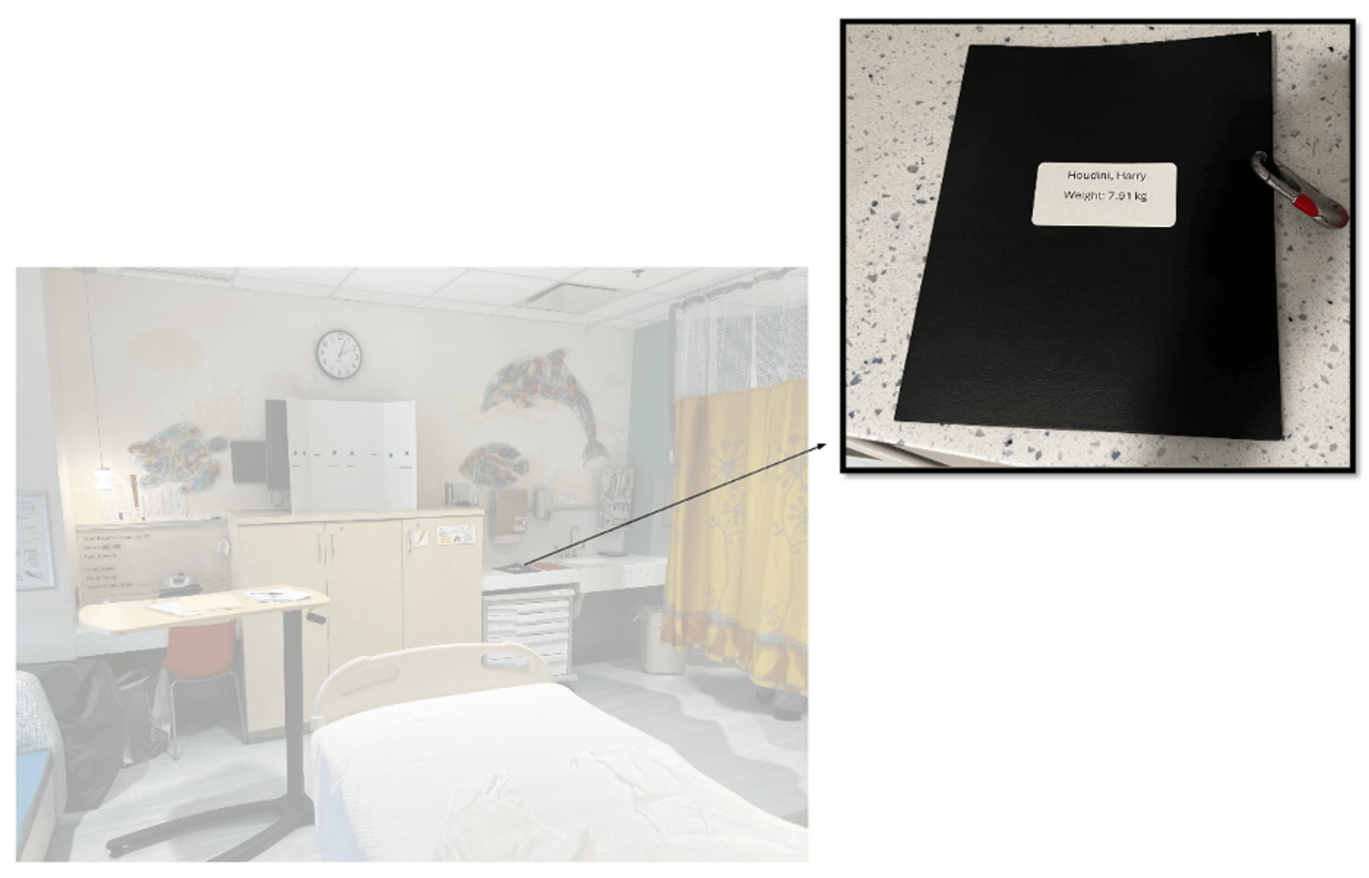
Puzzle 1: Simulated patient folder containing history of present illness (HPI). Weight on the front needed to unlock the folder.

**Figure 4:**
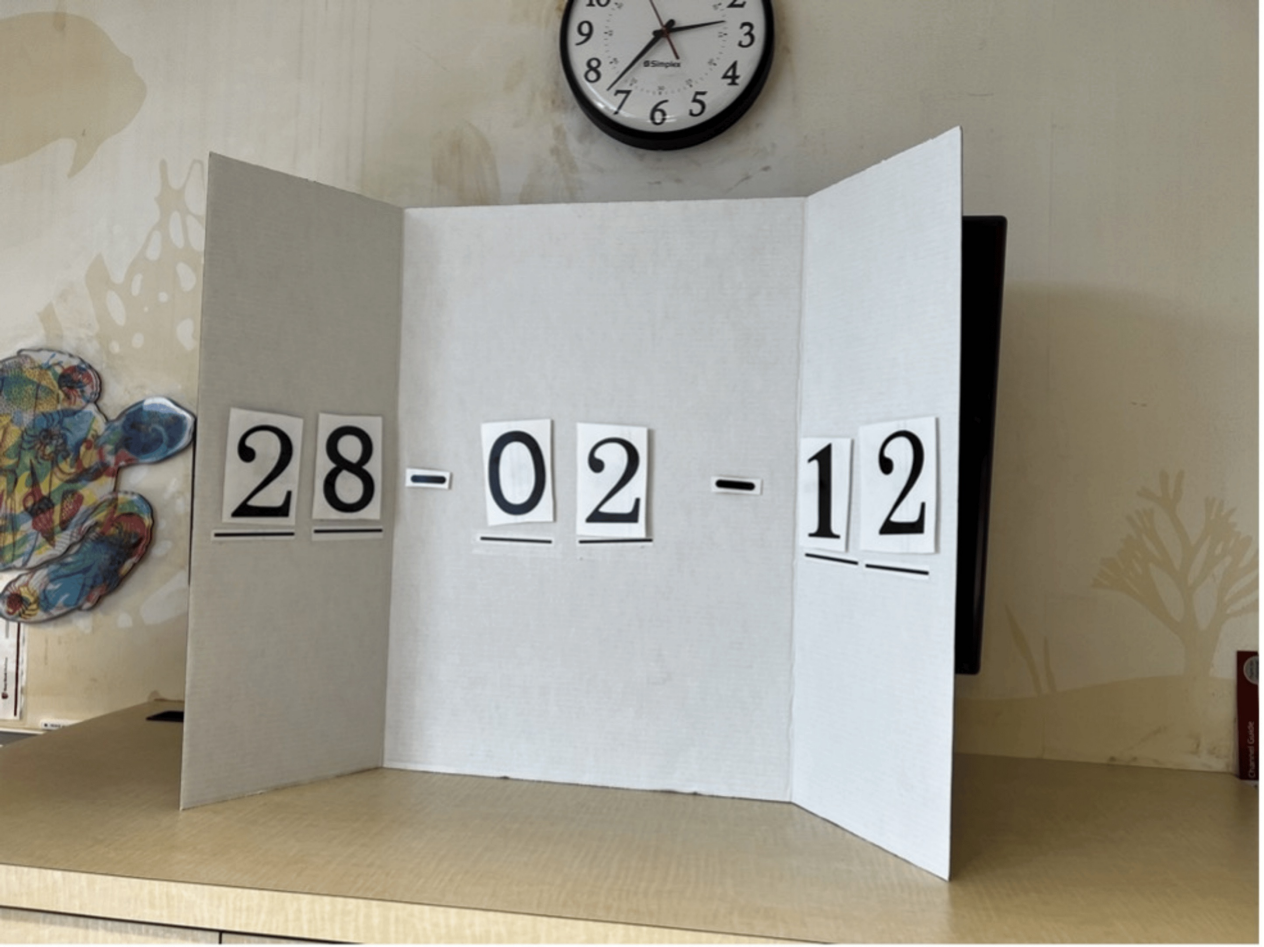
Master Code Board highlighting progress through the escape room as puzzles were solved.

**Figure 5:**
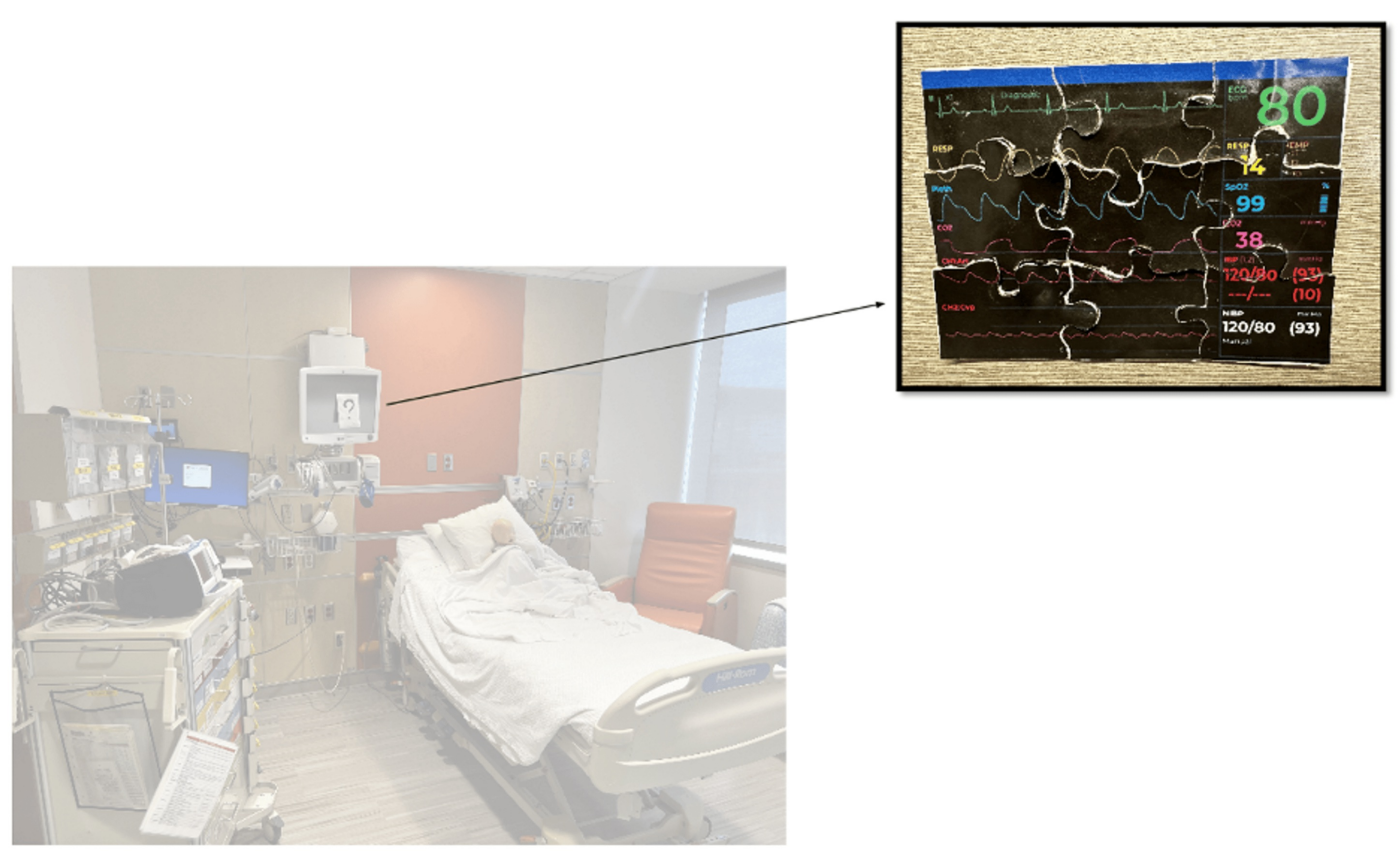
Puzzle 2: Jigsaw puzzle that needed to be solved to reveal updated patient vitals.

**Figure 6:**
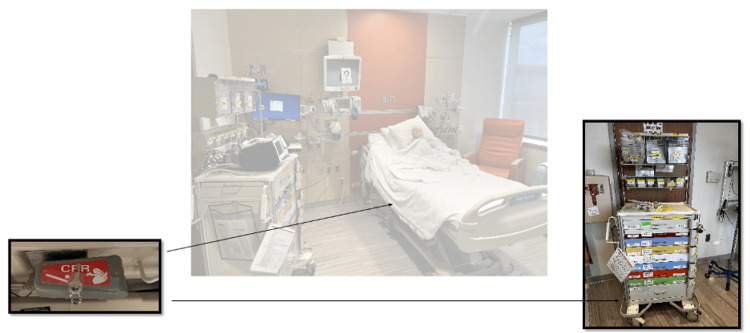
Puzzle 3: Cardiopulmonary resuscitation (CPR) lever that needed to be used to adjust bed, revealing key needed to unlock code cart to access bag-valve mask ventilation (BVM).

**Figure 7:**
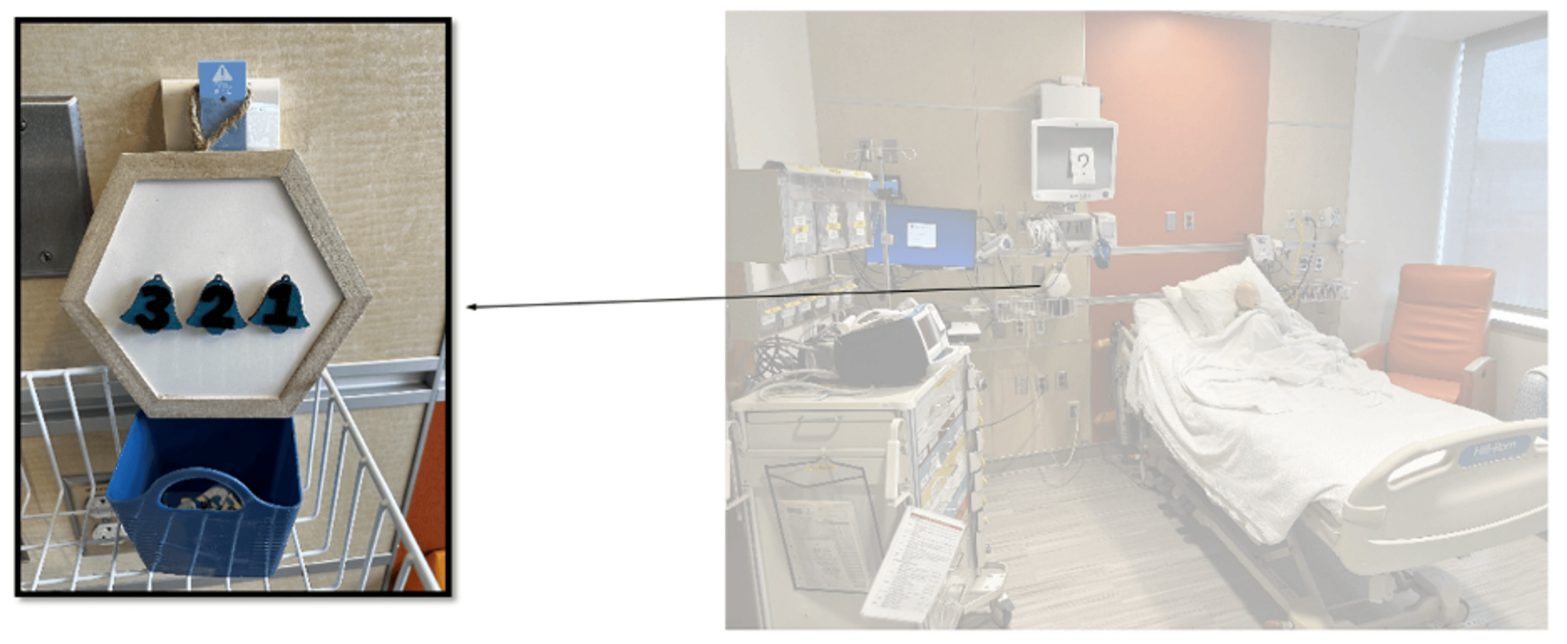
Puzzle 4: Number puzzle that needed to be arranged the sequence 3-2-1, our institutional number needed to call a "code blue."

**Figure 8:**
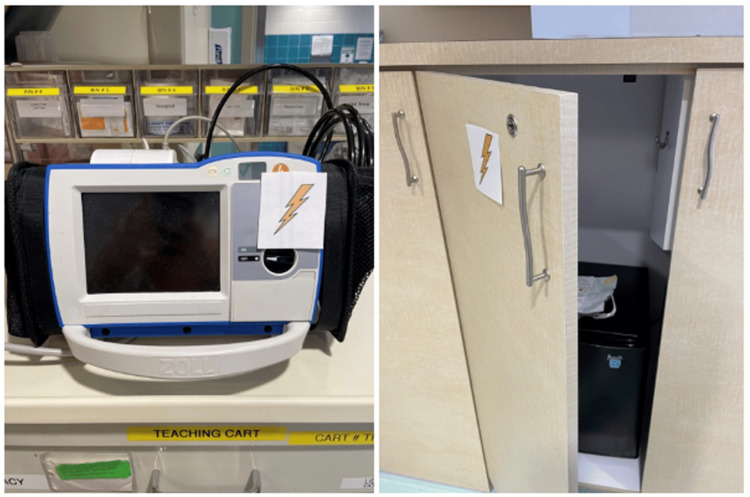
Puzzle 5: Lightning bolt on automated external defibrillator (AED) to match cabinet drawer containing the necessary connection pads.

**Figure 9:**
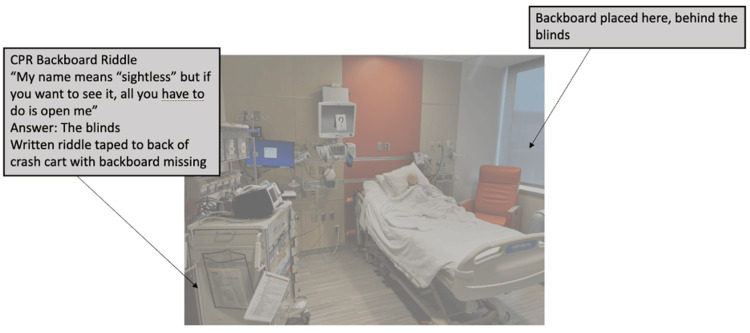
Puzzle 6: Riddle located in typical backboard location on crash cart which directed learner to hiding spot behind blinds.

The team entered the room to find an infant in respiratory distress without a parent or guardian at bedside. The infant was somnolent, in an upright hospital bed, with a working intravenous catheter (IV) in the arm, cardiac lead stickers on his chest that were disconnected from the monitor, and pulse oximetry ineffectively attached to him. To obtain additional patient information, learners needed to access a locked folder labeled with the patient’s name and three-digit weight (Figure 3). The weight was used as the code to the lock on the folder. When the three digits were entered correctly, the folder could be opened and revealed the patient’s history of present illness (HPI). Solving this first puzzle revealed the first number of a six-digit master code corresponding to a combination lock placed on the crash cart drawer containing epinephrine. Once the puzzle was solved, the facilitator would place this first number on a large poster board to keep track of the progression to the full six-digit combination (Figure 4). Simultaneously, learners discovered a blank cardiac monitor and recognized the need to obtain a more current set of vitals. An envelope containing a jigsaw puzzle hung from the monitor screen (Figure 5). Once solved, the monitor turned on and displayed updated vitals. Learners then identified hypoxia, tachypnea, and tachycardia prompting repositioning of the patient and initiation of bag-valve mask ventilation (BVM). In attempting to reposition the infant and lower the head of the bed, they would realize the bed was without power and required use of the CPR lever, which had a hidden key taped to it (Figure 6). This key opened the bottom drawer of the crash cart that contained the BVM. The learners could then begin BVM but the patient would continue to deteriorate. The team would recognize the worsening clinical status provided by the facilitators and agree to call a code blue, but would find the code bell to be nonfunctional until another three-digit puzzle was solved (Figure 7). Learners had a selection of digits to choose from with the answer being 3-2-1 (our institutional phone number used to call a code blue). These three numbers were attached to Velcro spots on a board hanging from the code blue bell, which activated an overhead code blue call. The patient’s heart rate would then drop below 60 beats per minute. This was identified by learners to be impending cardiac arrest. They would then initiate CPR as per the PALS algorithm. The next step was to attach pads to the patient, but the automated external defibrillator (AED) provided would not have connection pads. Learners then would need to match the lightning bolt symbol on the AED with the same symbol on a cabinet door containing the hidden pads (Figure 8). Once pads were attached and CPR was in progress, the team was prompted by the facilitator to continue chest compressions with the use of a backboard. In place of the backboard’s typical location on the back of the crash cart, a riddle was found (Figure 9). Solving the riddle prompted participants to look for the backboard behind window blinds. As each puzzle was solved (six in total) a number was added to the six-digit master code on the poster board at the front of the room. Once completed, this revealed the code to the lock on the medication drawer containing epinephrine as mentioned above. The team determined cardiac dosing of epinephrine using either the patient’s weight or the code sheet in the room. Once the correct dose of epinephrine was drawn and administered, the team was notified that they had successfully escaped the room.

Debriefing

As this simulation model was a new learning modality for our institution and its trainees, we focused the debrief partially on the structure of the escape room, perceived educational value for learners, and specific feedback for improvement. We also utilized a plus-delta model to ascertain from participants what they believed to have gone well and what could have been performed differently. Finally, learners were asked to reflect and share one takeaway regarding medical management and one regarding team building skills.

Assessment

We utilized a post-simulation survey to evaluate the effectiveness of the sessions (Appendix B). The survey inquired about each trainee’s educational experience with this new modality of learning. More specifically, it inquired about how the session improved medical knowledge, interpersonal communication, and how this novel form of simulation compared to prior experience with traditional mock codes. Our critical action checklist, which corresponded to solving all the six puzzles, was used to measure learner performance.

## Results

Session facilitators consisted of two attendings (one pediatric intensive care and one pediatric emergency medicine), two pediatric chief residents, and a pediatric intensive care nurse. Sessions were run by any combination of one or two of these individuals. A script was followed for the pre-brief, simulation flow, and debrief. The level of expertise in simulation varied among the four facilitators; however, all were required to be knowledgeable about the simulated patient case, escape room format, and how to conduct the debrief. All four facilitators were formally trained in simulation, with one facilitator being a board-certified healthcare simulation educator. Five escape room simulations were conducted. Post-simulation survey data was collected from four pediatric and medicine-pediatric residents ranging in post-graduate years from one to four, as well as five pediatric nurses. Over 20 learners participated in the five sessions; however, data was not collected in the initial sessions as the concept and design were being piloted and refined.

**Figure 10:**
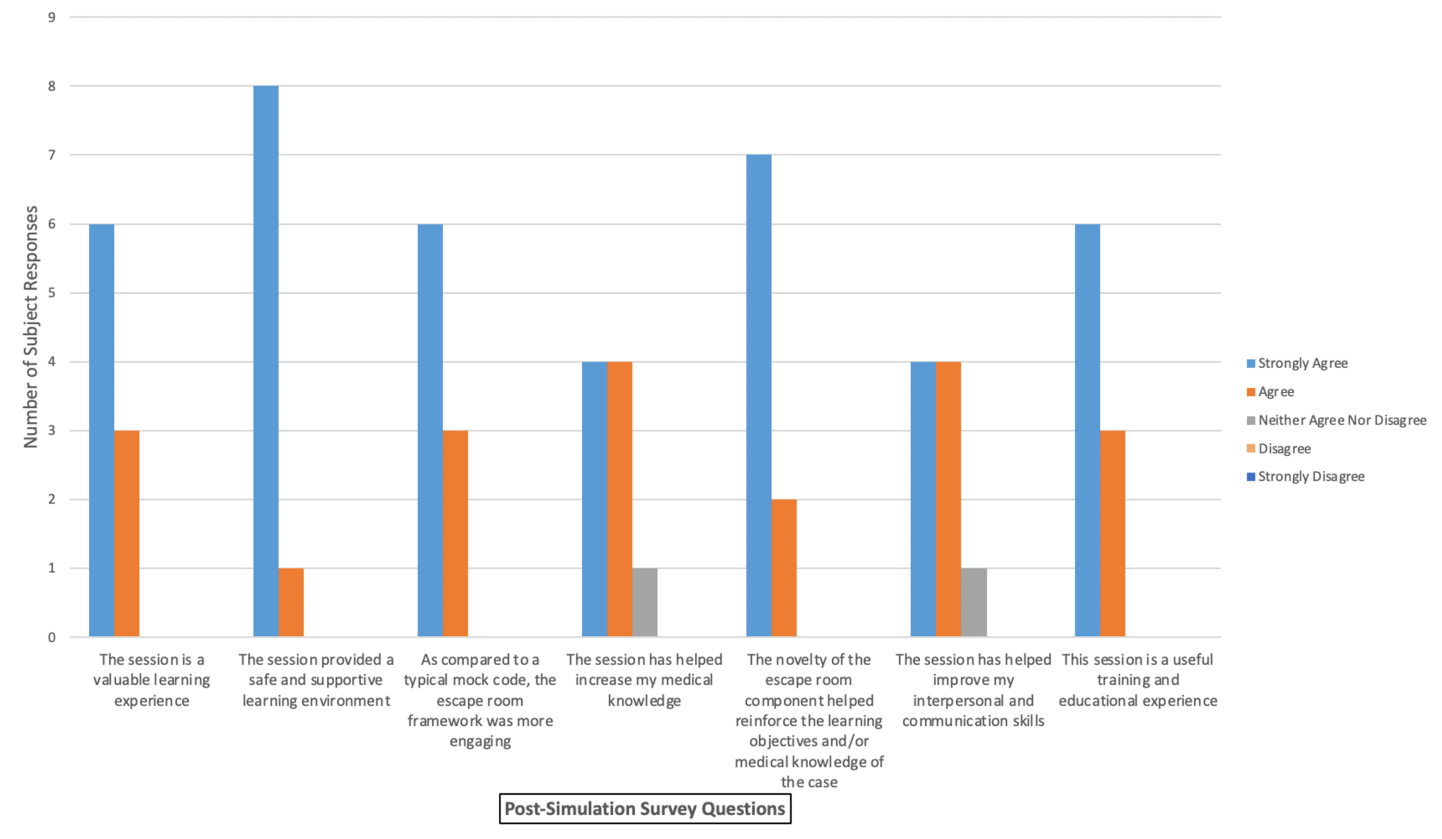
Post-Escape Room Survey

All the nine analyzed participants agreed or strongly agreed that this escape room simulation case provided a valuable learning and training experience, and a safe learning environment. The escape room was a more engaging form of simulation than a traditional mock code and helped reinforce learning objectives and knowledge of the case (9/9). Finally, 89% of the participants agreed or strongly agreed that the session increased medical knowledge and improved communication skills (Figure 10).

Regarding learner performance, this was measured by meeting marks on our critical action checklist. These marks corresponded to solving each puzzle embedded into the case. All five sessions resulted in learners solving the six puzzles; therefore 100% of groups satisfied all critical actions and escaped the room. These puzzles corresponded to learners demonstrating skills in escalation of care, BLS/PALS, and crisis resource management.

## Discussion

We incorporated escape room simulation into our pediatric residency curriculum as a novel way to engage residents and nurses in their own education, drawing from established principles of adult learning theory. More specifically, adults typically prefer learning environments that are self-directed, experiential, relevant, and offer a means to apply what was learned or practiced. The case was specifically developed to require learners to identify a decompensating young patient, escalate care, practice crisis resource management specific to our institution, and review principles of BLS and PALS. The escape room format forced trainees to think critically, problem solve, and communicate effectively all within a 30-minute time constraint. Participants reported that the session was a valuable learning experience and provided a supportive learning environment that was more engaging than a typical mock code. An increase in medical knowledge and improvement in communication skills were also endorsed subjectively from the learners.

With consecutive sessions, facilitators noted several opportunities for improvement. In fact, trainees were given the opportunity to provide immediate feedback following their participation. This was used to tailor subsequent sessions to better meet learner needs. The initial group struggled with providing medical care to the simulated patient while trying to solve puzzles, for example, providing BVM while collaborating with the team to solve the next puzzle. Thereafter, the study team introduced the concept of “ghost providers” for tasks that were “completed”. For instance, once a participant demonstrated effective bag mask ventilation or chest compressions, the facilitator announced a ghost provider would take over, freeing the learner to engage in other tasks. It is important to note that the ghost provider was not announced until the learner adequately demonstrated the skill to the facilitator so that learning objectives were still met. Another group noted that the pre-briefing placed an emphasis on the novel escape room framework such that aspects of patient care unrelated to puzzle progression were being neglected. The study team therefore modified the pre-briefing script and set up the patient room more authentically, that is, setting up the suction cannister or placing a code sheet in its typical location. Conversely, participants who were more familiar with escape rooms felt some of the puzzles were too simple, so we began to utilize “distractors” in subsequent sessions. These included numbers, symbols, or objects hidden around the patient room that resembled puzzle items but did not progress the task at hand as one might see in a commercial escape room. This added a level of difficulty to the case while increasing the fidelity of the simulation to create a more engaging environment for learners. We also added a code sheet unique to the simulated patient’s weight to encourage its use when calculating epinephrine dosing. Finally, we added puzzle 3 after our pilot session to draw the team’s attention to the CPR lever on our inpatient hospital beds.

The development of our escape room simulation was challenging as it was a new concept for all learners and for our institution. Participants sometimes interpreted tasks or approached puzzles in ways the study team did not anticipate. The lack of refinement in the earlier sessions resulted in more prompting from the facilitators, explanation, and debriefing. However, this was used to improve subsequent sessions as mentioned above. There are still several areas for improvement, including using better measures of clinical performance. We were able to report that all five teams solved the six puzzles, performed critical actions and escaped the room. In the future, we intend to incorporate more objective performance measures. For example, we plan to utilize a qCPR™ mannequin as the patient, which provides real-time metrics and feedback on the effectiveness of chest compressions and ventilation. Finally, we did not collect post-simulation survey data from the first three sessions as the focus of our pilot study was initially the development and implementation of the case itself and we acknowledge that the small sample size is a limitation of our study. Additional escape room cases and more trainees, both within and outside of our institution, are needed to further evaluate its effectiveness in pediatric medical education.

## Conclusions

Escape room simulation is a novel learning modality that can serve a role in pediatric medical education. It fosters a supportive learning environment and can bridge the knowledge gaps regarding rare pediatric emergencies that are critical for trainees to master. An example of such is pediatric resuscitation and cardiac arrest. Escape room simulation creates a unique domain for learners to practice the medical management of the critically ill child. It is reported to be a positive educational experience overall, increase medical knowledge, improve communication, and preferred over traditional mock code formats.

## Figures and Tables

**Figure 11:**
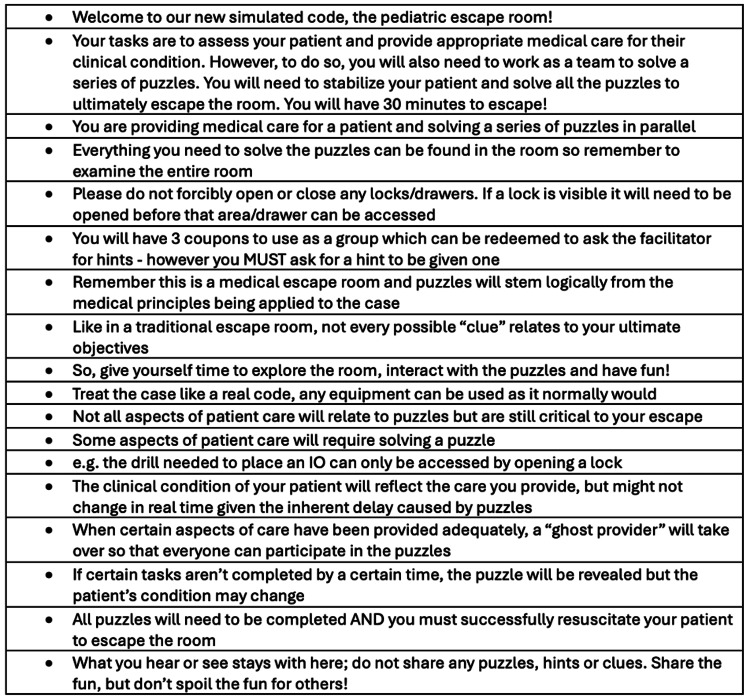
Appendix A: Pre-Brief Script

## Appendices

Appendix B: Post-simulation survey

Date:

Current Year of training?

o      PGY 1

o      PGY 2

o      PGY 3

o      PGY 4

o      R.N.

o      Nursing Student

o      MS 3

o      MS 4

o      Other _______

Where did your simulation take place?

o      Simulation Lab

o      In-Situ

o      Other _________

Learning objectives were available prior to the session:

o      Strongly Agree

o      Agree

o      Neither Agree nor Disagree

o      Disagree

o      Strongly Disagree

The learning objectives were met:

o      Strongly Agree

o      Agree

o      Neither Agree nor Disagree

o      Disagree

o      Strongly Disagree

Have you ever done an escape room before?

o      Yes

o      No

The session is a valuable learning experience:

o      Strongly Agree

o      Agree

o      Neither Agree nor Disagree

o      Disagree

o      Strongly Disagree

The session provided a safe and supportive learning environment:

o      Strongly Agree

o      Agree

o      Neither Agree nor Disagree

o      Disagree

o      Strongly Disagree

I have participated in a typical rapid-cycle-debrief mock code previously:

o      Yes

o      No (if selected skip the next question)

As compared to a typical mock code, the escape room framework was more engaging:

o      Strongly Agree

o      Agree

o      Neither Agree nor Disagree

o      Disagree

o      Strongly Disagree

o      N/A

The session has helped increase my medical knowledge:

o      Strongly Agree

o      Agree

o      Neither Agree nor Disagree

o      Disagree

o      Strongly Disagree

The novelty of the escape room component helped reinforce the learning objectives and/or medical knowledge of the case:

o      Strongly Agree

o      Agree

o      Neither Agree nor Disagree

o      Disagree

o      Strongly Disagree

The session has helped improve my interpersonal and communication skills:

o      Strongly Agree

o      Agree

o      Neither Agree nor Disagree

o      Disagree

o      Strongly Disagree

This session is a useful training and educational experience:

o      Strongly Agree

o      Agree

o      Neither Agree nor Disagree

o      Disagree

o      Strongly Disagree

Do you have any comments or suggestions?

Do you have suggestions for future training sessions?

I feel comfortable in a pediatric code:

o      Strongly Agree

o      Agree

o      Neither Agree nor Disagree

o      Disagree

o      Strongly Disagree

I want to get more experience in a pediatric code:

o      Strongly Agree

o      Agree

o      Neither Agree nor Disagree

o      Disagree

o      Strongly Disagree

I know how to communicate well in a pediatric code:

o      Strongly Agree

o      Agree

o      Neither Agree nor Disagree

o      Disagree

o      Strongly Disagree

I know what my role is in a pediatric code:

o      Strongly Agree

o      Agree

o      Neither Agree nor Disagree

o      Disagree

o      Strongly Disagree

This simulation offers a good opportunity for training non-technical skills (e.g. teamwork, knowledge, etc.)

o      Strongly Agree

o      Agree

o      Neither Agree nor Disagree

o      Disagree

o      Strongly Disagree
